# An Atypical Metastasis of Follicular-Type Adenocarcinoma of the Thyroid Gland to Thumb

**DOI:** 10.1155/2011/735789

**Published:** 2011-12-29

**Authors:** Gazi Huri

**Affiliations:** Department of Orthopaedic and Traumatology Surgery, Cukurova University, 01330 Adana, Turkey

## Abstract

Bone metastasis in the hand is rare. The etiology of metastatic hand cancers is different from other bones. Bronchogenic carcinoma is the most common primary tumor metastasis to hand. In this paper a rare case of thumb metastasis from “follicular-type carcinoma” of the thyroid is presented.

## 1. Introduction

Incidence of metastasis of malignant neoplasms in the extremities is rare, and the hands contribute to about 0.007–0.1% of this incidence [1–4]. Hand metastasis may develop from lung, breast, kidney, and gastrointestinal cancers. The distal phalanx is the most commonly involved site for hand metastases. In this paper a rare case of thumb metastasis from “follicular-type carcinoma” of the thyroid is presented. Written informed consent of the patient for printed and electronic publication for case report was obtained.

## 2. Case Report

**Figure 1:**
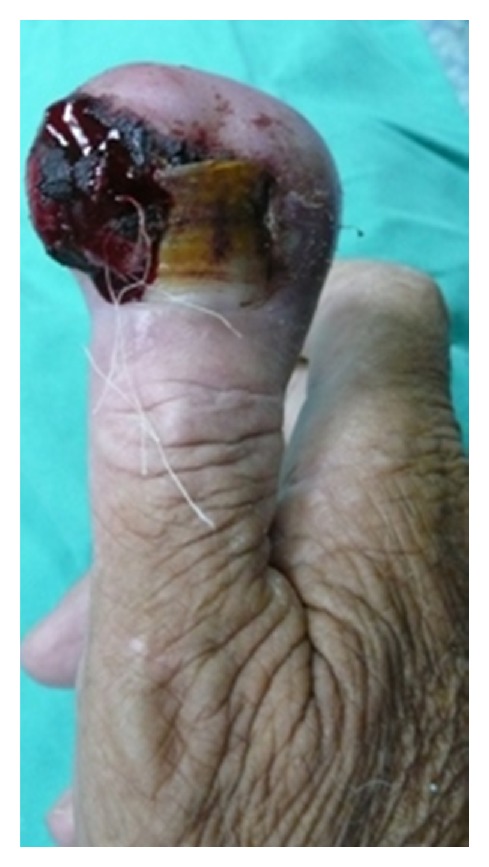
Swelling and hemorrhaging at distal part of the right thumb.

An eighty-four-year-old right-hand-dominant man, who had a history of thyroidectomey in 2007, presented with a 2-month history of increasing pain and swelling at the distal part of the right thumb (Figure 1). He had no history of hand trauma and no previous history of joint pain. There were no abnormal results in complete blood count (CBC), erythrocyte sedimentation rate (ESR), and C-reactive protein values.

**Figure 2:**
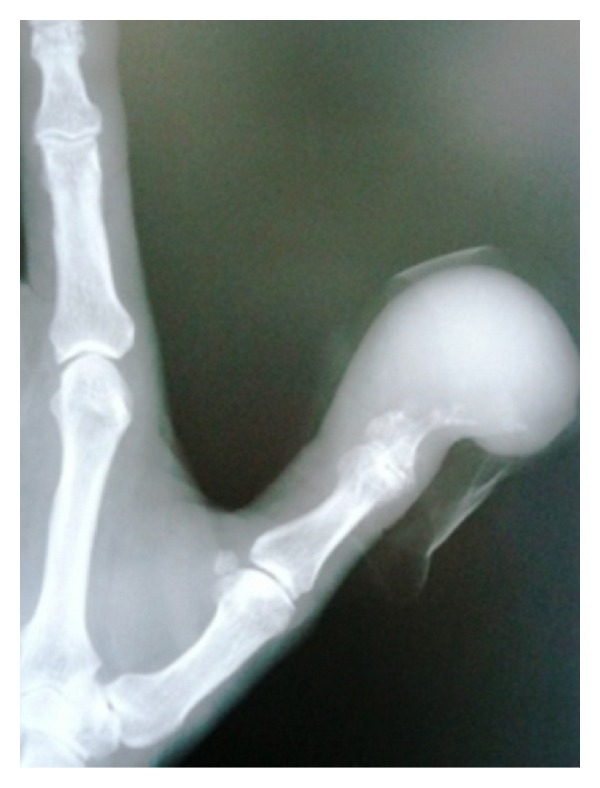
Radiological view of thumb: soft-tissue swelling and lysis of the distal phalanx of thumb.

**Figure 3:**
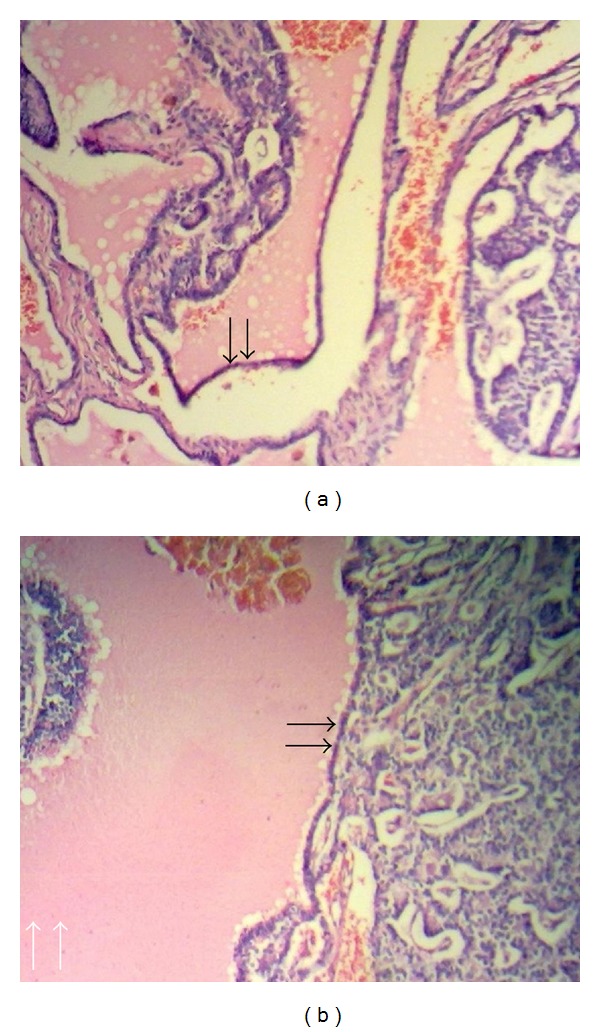
(a) Histopathological specimen of metastatic follicular-type carcinoma of the thyroid gland into the skin and soft tissue of amputated thumb. (b) Well-differentiated epithelium is clearly distinguished (black arrows) with follicular development and colloid (white arrows).

Radiological evaluation demonstrated a soft-tissue swelling and lysis of the distal phalanx of thumb, compatible with infection priority or metastatic disease (Figure 2). The bone scan revealed no other pathologic lesion. The patient underwent an incisional biopsy from right thumb. Both microbiologic and histopathologic examinations of the specimen were performed. While microorganisms were not isolated from the specimen, in histopathological analysis, well-differentiated epithelium is clearly distinguished (black arrows) with follicular development and colloid which confirmed diagnosis of metastatic follicular-type carcinoma of the thyroid gland (Figures 3(a) and 3(b)).

Afterwards, the patient underwent proximal phalangeal amputation of the right thumb under local anesthesia. The postoperative course was normal and the wound healed without complication.

## 3. Discussion

Metastatic malignancies of the hand are rare and they usually develop from lung, breast, and kidney tumors. In this paper, a rare case of thumb metastasis from “follicular-type carcinoma” of the thyroid is presented. Isolated cases of thumb metastasis from several origins have been described in the literature [5–7]. Kerin in 1983 published the most recent review of 163 hand metastases in the world literature. According to this paper, the most common site of hand metastases is the distal phalanges (51%), followed by the carpal bones (29.5%) and the metacarpals (27.6%) [2].

Even though hand tumors can be determined easily because of their localization, diagnosis is usually difficult. Symptoms and findings, including swelling, erythema, and pain as the most frequent symptoms occurred in infection, osteomyelitis, rheumatoid arthritis, and gout should be kept in mind as differential diagnosis [8, 9]. Bone scans, microbiological and histopathological examinations, as well as meticulous and radiological investigations must be done for definitive diagnosis. Treatment must be planned according to the results of these evaluations.

Treatment options of these lesions are dependent on the status of the patient, primary origin of the metastases, and localization. It is well known that metastatic hand tumors appear at a late stage of primary tumor and survival is usually less than 6 months; therefore the treatment is usually palliative [10, 11]. Chemotherapy, radiotherapy, and surgical approaches are options for treatment. Both chemotherapy and radiotherapy may be sufficient for reducing tumor mass and relieve pain in patients with multiple or inoperable lesions. Surgical treatment depends on the localization. Excision, amputation, ray resection, or curretage may be alternative methods especially for small lesions [9]. In the presented case, because of the localization of the lesion, patient status, and age, wide resection (amputation) as a treatment option after insicional biopsy was preferred.

One other case of metastatic thyroid carcinoma to the hand has been reported in 1976 by Uriburu et al. on metastatic tumor of ring finger from tyroid carcinoma in the literature [12]. This case is the second case reporting the metastatic tumor of a finger from thyroid carcinoma.
